# Ovarian Dermoid Cysts: Does Size Matter When it Comes to Laparoscopic Surgery?

**DOI:** 10.7759/cureus.83999

**Published:** 2025-05-13

**Authors:** Bashayer Almaazmi, Anjala Nizam, Huda Ahmed, Arnaud Wattiez, Haroutyoun Margossian

**Affiliations:** 1 Obstetrics and Gynecology, Latifa Women and Children Hospital, Dubai, ARE; 2 Obstetrics and Gynecology, Al Qassimi Women’s and Children’s Hospital, Sharjah, ARE

**Keywords:** chemoperitonitis, laparoscopy, oophorectomy, ovarian cyst, ovarian torsion, ultrasound

## Abstract

Objectives

This study aims to determine the sensitivity of preoperative imaging in detecting ovarian dermoid cysts. It aims to explore cyst size as a determinant of surgical approach, intraoperative surgical complications such as cyst spillage/leak, and the postoperative complication of chemical peritonitis. It delves into the discussion of torsion, determining the sensitivity of vomiting as a presenting symptom and the accuracy of preoperative Doppler ultrasound in detecting torsion by correlating radiological diagnosis with the intraoperative diagnosis.

Methods

Eighty-seven cases were retrospectively extracted from the Department of Obstetrics and Gynecology at Latifa Women and Children Hospital, Dubai, United Arab Emirates, between November 2017 and February 2020.

Results

Of the 87 cases, 71 underwent transvaginal or transabdominal ultrasonography, 17 underwent CT scan, and 10 underwent MRI. Nine cases underwent both ultrasound and CT scan. Eight cases underwent ovarian cystectomy or oophorectomy via laparotomy, whereas 79 cases underwent ovarian cystectomy or oophorectomy via laparoscopy. Torsion was suspected in patients presenting with vomiting. Preoperative Doppler ultrasound was used to detect the presence of torsion, which was confirmed intraoperatively.

Conclusions

Ultrasound is 70% sensitive, with an 81% positive predictive value, in detecting dermoid cysts. Cyst size affects the choice of surgical approach (p<0.02), with larger cysts undergoing laparotomy, although laparotomy has a higher risk of an intraoperative leak. Cyst size did not influence the risk of leak (p<0.59), and no cases of chemical peritonitis were recorded following effective intraoperative peritoneal washouts. Vomiting as a presenting complaint indicates the presence of torsion (p<0.001), with a sensitivity of 70% and specificity of 80.6%. Almost 90% of torsion cases were identified preoperatively via ultrasound Doppler and confirmed intraoperatively.

## Introduction

The most common type of ovarian germ cell tumor is a teratoma. Teratomas are further categorized into mature teratomas (cystic, solid, monodermal, or mature with malignant transformation) and immature teratomas (high-grade or low-grade) [1]. Ovarian dermoid cysts and mature cystic ovarian teratomas are often referred to interchangeably. While their imaging characteristics are very similar, they differ histologically. Dermoids are made up solely of ectodermal elements, whereas teratomas contain components from all three germ layers: ectoderm, mesoderm, and endoderm. For simplicity, both terms are addressed together in this article, as much of the existing literature treats them as one entity.

Mature cystic teratomas (MCTs) account for at least 70% of benign ovarian tumors in women less than their third decade of life and 50% of pediatric tumors [2]. MCTs arise from totipotent cells in the ovary that transform into differentiated ectodermal (e.g., skin, hair follicles, and sebaceous glands), mesodermal (e.g., muscle), and endodermal (e.g., gastrointestinal and pulmonary) tissues [3]. Only 10-17% of MCT cases are bilateral, and secondary malignant transformation is a rare but well-known phenomenon, with squamous cell carcinomas accounting for 80% of ovarian teratoma malignant transformations [4]. Risk factors for such transformation include increased age (>45 years), tumor diameter of >10 cm, rapid tumor growth, and specific findings on imaging such as low-resistance intratumor flow on Doppler ultrasonography [5].

Women with MCTs are mostly asymptomatic and are usually found incidentally during a routine examination or via imaging. A review of 517 cases noted that 60% of patients were asymptomatic at the time of diagnosis [6]. The most common symptom, if present, is abdominal pain, followed rarely by palpable abdominal mass, constipation, nausea, vomiting, and anorexia [7]. However, 3-4% of women present with acute pelvic pain, usually due to ovarian torsion, the risk of which is correlated with a size of more than 5-6 cm. Emergency surgery is indicated in this cohort. Ovarian untwisting can be considered in adolescents where recovery of function is possible [8,9]. In general, the surgical management of MCT is ovarian cystectomy via laparoscopy. Oophorectomy is the standard of care in postmenopausal and perimenopausal women; in women who present with multiple cysts in the same ovary; in women who present with very large MCTs where there is no chance for ovarian preservation [10]. Surgical excision also provides the definitive diagnosis of MCT.

Pelvic ultrasonography is the most basic preoperative imaging modality currently in use to diagnose adnexal masses. Features such as the Rokitansky nodule, tip of the iceberg sign, dot-dash sign, and fat-fluid sign highlight a wide variety of echo combinations, leading to difficulty in providing a definitive diagnosis. However, some features are more characteristic than others, and when two or more are present, this will increase the positive predictive value of sonography as a diagnostic test [11]. CT scan is noted for its excellent sensitivity (93-98%) due to its ability to detect fat and subtle calcifications in the MCT. When it comes to MRI, many studies have underscored the superiority of contrast-enhanced MRI when compared to ultrasound in characterizing adnexal mass lesions [12,13].

## Materials and methods

This study was conducted in the Department of Obstetrics and Gynecology at Latifa Women’s and Children’s Hospital, Dubai. After obtaining institutional review board approval, a total of 300 cases were extracted via "SALAMA LOG CODE" by using the Current Procedural Terminology (CPT®) code for "Ovarian Cystectomy and Oophorectomy." Following this, using the International Classification of Diseases (ICD-9) diagnostic code for "Dermoid" or "Cystic Teratoma" as the diagnosis within imaging reports or histopathology reports between November 2017 and February 2020, a total of 87 cases were extracted and analyzed.

The medical records of these patients were retrospectively reviewed and the following parameters were collected: date of surgery, patient nationality, age, case urgency, gravida, parity, patient's height and weight, BMI, past medical history, past surgical history, past gynecological history, family history, symptoms at presentation (dysmenorrhea, nausea, abdominal pain, dyspareunia, vomiting, asymptomatic, other), ultrasound report with Doppler flow, CT report, MRI report, blood tests for tumor markers (AFP, CA-125, CA 15-3, CA 19-9, CEA), surgical notes, type of surgery, and final histopathology report.

Preoperatively, all patients underwent bedside ultrasound; however, for this study, only reports by certified radiographers were used. Among the study population, 71 patients underwent ultrasound imaging. Details regarding the side of the cyst (left versus right), size of the cyst (in cm), volume, heterogenous versus homogenous appearance, presence of septations, evidence of solid-cystic separation, presence of acoustic shadowing, presence of calcification, cyst complexity, cyst hyperechogenicity, evidence of torsion on Doppler study, report on differential diagnosis, and type of vascularity via Doppler flow were recorded and analyzed. Seventeen patients underwent investigation via CT scan; their details regarding the side, size, presence of calcifications, cyst features, presence of septations, homogenous versus heterogeneous components, and differential diagnosis were collected. Ten patients underwent investigation by MRI, and similarly, the information regarding the side of the cyst, size, presence of septations, evidence of bone density, and differential diagnosis was also collected and analyzed. Nine patients underwent both ultrasound and CT scan. Imaging yielded a positive result if the differential diagnosis at the end of the imaging report included the words: "Dermoid" or "Cystic Teratoma." The gold-standard histopathology report yielded a positive result if it entailed the words "Dermoid Cyst" or "Cystic Teratoma" within its diagnosis.

The type of surgery patients underwent (laparoscopic cystectomy, laparoscopic oophorectomy, laparotomy for cystectomy, laparotomy for oophorectomy) was recorded. Postoperative surgical notes were used to extract whether there was any evidence of torsion or not, and whether there was any evidence of intraoperative leakage or cyst rupture. Patients who presented with bilateral ovarian cysts were recorded as two separate entries. Patients who presented with multiple unilateral cysts were recorded as one entry, and those with information about the biggest cyst were selected to represent the case. One patient in this study group presented with ovarian cyst torsion on admission, and a laparoscopic detorsion of the ovary was performed. However, a week later, she was readmitted again with torsion and managed via laparoscopic oophorectomy. This was presented and analyzed as two separate entries.

## Results

The mean age of our patients in the study group was 27.90 years (SD ± 7.86), the youngest being 13 and the oldest being 44. In regard to BMI, 4.5% were underweight, 39.8% were normal, 34.1% were overweight, and 21.6% were obese. Of the 71 patients who underwent ultrasonography, 54 patients were given a preoperative diagnosis of a "dermoid cyst." The preoperative sensitivity of ultrasonography in diagnosing dermoid cysts was highlighted as 70.5%, with a positive predictive value of 81%. Other diagnoses, revealed by the histopathology report, were of spindle cell tumor, benign mucinous cystadenoma, benign hemorrhagic corpus luteal cyst, hemorrhagic ovarian follicle, normal tissue, benign endometriotic tissue, or benign simple cyst.

The decision for laparoscopy versus laparotomy was personalized based on the patient’s clinical presentation, the appearance of the cyst on imaging, and whether there is concern for malignancy or other complications. In general, cysts above 10 cm were encouraged to undergo laparotomy rather than laparoscopy. Patients who underwent laparoscopy (including laparoscopic oophorectomy or laparoscopic cystectomy) had a mean cyst size of 7.37 cm and a median of 6.70 cm (SD ± 3.71). However, patients who underwent exploratory laparotomy had a mean cyst size of 11.62 cm and a median of 12.45 cm (SD ± 2.98). The Mann-Whitney U test was used for analysis due to the skewed number variables. It showed that the size of the cyst played a significant role (p<0.02) in choosing the surgical approach, as the laparotomy approach was used for cysts larger than 12 cm. 

In reference to the patient's BMI and size of the cyst, we explored whether or not these parameters influenced our choice of surgical approach; laparoscopic or laparotomy. The mean BMI of patients undergoing laparoscopic surgery was 26.570 (SD ± 5.58), while those who underwent laparotomy had a BMI of 26.95 (SD ± 6.70). Mann-Whitney U test showed that the BMI did not have any influence on choosing our surgical approach and how to perform her ovarian cyst surgery via laparoscopy or laparotomy (p<0.798).

Cyst rupture and intraoperative surgical leak are common concerns, especially if the final histopathology confirms a final diagnosis of malignancy. Therefore, we looked at the cyst rupture (leak) rate during our surgeries, whether it occurred more with laparoscopy or laparotomy, and whether the cyst size influenced its occurrence. Cysts that leaked had a mean size of 7.60 (SD ± 3.94), while cysts that did not leak had a mean size of 7.86 (SD ± 3.58). The size of the cyst did not influence its risk of intraoperative leak or rupture, as highlighted by the Mann-Whitney U test (p<0.59). We also looked into the occurrence rate of an intraoperative leak during laparoscopy versus laparotomy and found a 69% intraoperative cyst leak with laparoscopic ovarian cystectomy, in contrast to a 100% spillage rate in patients undergoing exploratory laparotomy (Table 1). The Fisher's exact test was used, and no statistical significance was found due to the small sample size (eight cases) of laparotomies in our study (p<0.315).

**Table 1:** Intraoperative leak and type of surgical intervention n, number of cases

Type of surgical intervention	Intraoperative leak	
	Present (n(%))	Absent (n(%))
Laparoscopic	55 (68.8)	25 (31.3)
Laparotomy	5 (100.0)	0 (0.0)
Total	60 (70.6)	25 (29.4)

We also looked into the accuracy of torsion diagnosis via preoperative Doppler ultrasound study (Table 2). Approximately 90% of the cases identified preoperatively via Doppler ultrasound were confirmed as torsion during surgery. However, only 87.5% of the cases identified as no torsion via preoperative Doppler ultrasound did not have evidence of torsion intraoperatively. When the ultrasound Doppler report highlighted "possible torsion diagnosis," then the chance of finding torsion intraoperatively was only 44.4%. Ultrasound Doppler proved to have a significant role in correctly identifying the presence of torsion preoperatively (Chi-square test showed p<0.001). 

**Table 2:** Doppler ultrasound detection of ovarian torsion based on vascularity (A) and intraoperative findings of ovarian torsion (B) n, number of cases

Presence of ovarian torsion on Doppler ultrasound	A. Detection of vascularity using Doppler ultrasound			B. Intraoperative findings of ovarian torsion	
	Detected (n(%))	Not Detected (n(%))	Inconclusive (n(%))	Present (n(%))	Absent (n(%))
Torsion present	4 (40.0)	6 (60.0)	0 (0.0)	9 (90)	1 (10)
No evidence of torsion	22 (73.3)	7 (23.3)	1 (3.3)	5 (12.5)	35 (87.5)
Possible torsion	14 (82.4)	3 (17.6)	0 (0.0)	8 (44.4)	10 (55.6)
Total	40 (70.2)	16 (28.1)	1 (1.8)	46 (67.6)	68 (100)

We also looked at the association between the presence of torsion and the patient’s symptoms such as type, onset, and pattern of pain, nausea, and vomiting. Similar to other studies, our study confirmed that only vomiting is an indicator of the presence of torsion (Chi-square test showed p<0.001). In our study, 70.6% of patients who presented with vomiting had confirmed torsion during their surgery (Table 3). Our analysis showed that the presence of vomiting on admission was predictive of torsion, with a sensitivity of 70% and specificity of 83.1%. 

**Table 3:** Vomiting as a presenting symptom of ovarian torsion n, number of cases

Vomiting	Ovarian torsion	
	Present (n(%))	Absent (n(%))
Yes	12 (70.6)	5 (29.4)
No	12 (16.9)	59 (83.1)
Total	24 (27.3)	64 (72.7)

## Discussion

The sensitivity reported in this study for the use of ultrasonography in detecting MCT was 70.5%. This is comparable to studies with similar sample sizes, such as that of Hanife et al. in which 119 cases were used, reporting a sensitivity of 67.2%. Also, de Kroon et al. found a sensitivity of 80% in a sample of 99 cases [14,15]. Studies have continuously underlined the importance of ultrasound features in detecting MCTs. Features such as echogenic nodules, foci, or lines were highlighted in a study by Mais et al. in which the sensitivity was 58-99% for a sample of 659 cases. In a 2014 study by Tekin et al., 40 patients highlighted a sensitivity of 81.8%, stating that acoustic shadowing, the presence of echogenic foci or ridges formed by the hair, can be seen at high rates (between 60% and 90%) and contributes a sense of pattern recognition to diagnostic criteria [16,17]. This is an important factor as to why the specificity of our study proved to be inherently low (close to 0%), alluding to the small sample size. Still, the lack of detailed diagnostic reporting and the use of a long list of differential diagnoses can underline a lack of decision-making precision and accuracy or radiographer expertise in need of honing.

This study falls short in categorizing the type of ultrasonography patients had undergone (whether they were diagnosed via transvaginal (TVS) versus transabdominal (TAS) ultrasound), and this can be a reason for misdiagnosis, lack of radiological precision, or radiographer interpretation difficulty. As highlighted by Niazi et al. in their retrospective study of 200 patients, TVS is superior to TAS, boasting a sensitivity of 96% and a specificity of 89% when compared to a sensitivity of 91% and a specificity of 83% in TAS. The comparison proved to be statistically significant with a p<0.05 [18].

The incidence of intraoperative leak of cysts undergoing cystectomy was also analyzed in the current study. It was found that 69% of the cases managed via laparoscopy leaked. This is also reflected by Nezhat et al., who conducted a 10-year study on the management of dermoid cysts on a sample size of 81 patients [19]. They recorded a spillage rate of 48% per patient and a total spillage rate of 42%. They further highlighted that spillage could be attributed to the method of extraction, with 60% of spills occurring with aspiration and using a trocar sleeve without an endo-bag, 44% for removals via colpotomy, and 13.6% for removals with an endo-bag. Spillage was not correlated with the size of the cyst, as highlighted by our study, where the p-value of the association between spillage and size is p<0.54, highlighting statistical insignificance. This highlights the thought that the size of the cyst should not deter a skilled surgeon from taking a laparoscopic approach. This is also emphasized by a case report where large ovarian cysts were extracted via laparoscopy and in a randomized control trial conducted by Yuen et al. [20,21].

The current study compared the results of laparoscopy with laparotomy on benign ovarian masses with a diameter of more than 10 cm on ultrasonography. Their results highlighted that there was no increase in operating time and that the frequency of leak and spillage is almost equal. However, they noted a significant reduction (OR 0.34, 95% CI 0.13 to 0.88) in postoperative morbidity, pain, and recovery associated with the laparoscopic arm. However, Nezhat et al. highlighted that spillage rates in laparoscopic surgeries exceeded those of laparotomy, with percentages coming to 15-100% and 4-13%, respectively [19]. This is in contrast to our study, in which a leak rate of 100% was found in patients undergoing exploratory laparotomy. However, this could be attributed to the very small sample size of eight patients in the exploratory laparotomy arm. Another important notation that we need to emphasize is that despite the fact that 69% of cases leaked in our study, none of those patients presented with the signs and symptoms of acute chemical peritonitis. This can be attributed to the thorough peritoneal washings performed in surgery [22].

Ovarian torsion, partial or complete rotation of the ovary around its peduncle, leading to obstruction of venous or arterial flow, is a well-known sequela of ovarian cysts, including MCTs. The use of Doppler ultrasound is well documented in detecting torsion, where the presence of flow can be a determinant in diagnosis. However, the presence of color flow on Doppler imaging does not exclude the possibility of torsion but suggests that there is a chance of the ovary being viable, especially if a central flow is detected, while the absence of flow can suggest a lack of viability [23]. As with this study, 90% of torsion cases were detected preoperatively via Doppler ultrasound.

Nausea and vomiting occur in at least 85% of all adnexal torsion cases [24]. A study by Damigos et al. highlighted the scoring system used to identify adnexal torsion in women. The criteria involve unilateral abdominal pain, pain duration of more than 8 hours, vomiting, and absence of leucorrhoea/metrorrhagia and ovarian cyst of more than 5 cm on ultrasound [25]. The same study also demonstrated that vomiting held an adjusted OR of 7.9 (2.3 to 27), underlining its role as a predictive tool that can alert physicians to the occurrence of torsion and stratify patients into low or high-risk groups accordingly [25].

## Conclusions

This retrospective study underscores the significance of imaging, clinical presentation, and cyst characteristics in the diagnosis and surgical management of MCTs at our tertiary care center. MCTs represented the most common ovarian germ cell tumor encountered, predominantly affecting women in their reproductive years, with a mean age of 27.9 years. While widely used, ultrasound demonstrated a moderate sensitivity (70.5%) in diagnosing MCTs, with key features such as calcification and complex echogenicity improving diagnostic accuracy. CT and MRI offered enhanced diagnostic clarity, particularly in complex or equivocal cases.

Surgical management was significantly influenced by cyst size, with laparotomy primarily reserved for cysts >12 cm (p<0.02), whereas laparoscopy was the preferred approach for smaller cysts. BMI did not significantly impact the choice of surgical method. While intraoperative cyst leakage was common, occurring in both laparoscopic and open approaches, cyst size did not significantly predict rupture risk. Torsion was a critical surgical emergency identified in a subset of patients, and Doppler ultrasonography demonstrated high diagnostic reliability, especially when combined with clinical signs such as vomiting. Vomiting on presentation emerged as a statistically significant predictor of torsion (sensitivity 70%, specificity 83.1%; p<0.001). Overall, this study emphasizes the importance of an individualized, evidence-based approach to managing ovarian dermoid cysts, balancing accurate preoperative diagnosis, fertility preservation, and timely surgical intervention, particularly in suspected torsion cases.
